# Healthcare Students' Perceptions of the Quality of the Clinical Learning Environment in Morocco: A Cross-Sectional Study

**DOI:** 10.1155/2024/7849334

**Published:** 2024-05-21

**Authors:** Khadija Guejdad, Ali Ikrou, Camilla Strandell-Laine, Redouane Abouqal, Jihane Belayachi

**Affiliations:** ^1^Laboratory of Biostatistics, Clinical and Epidemiological Research, Faculty of Medicine and Pharmacy, Mohammed V University, Rabat, Morocco; ^2^Higher Institute of Nursing Professions and Health Techniques, Agadir, Morocco; ^3^Novia University of Applied Sciences, Turku, Finland; ^4^Lovisenberg Diaconal University College, Oslo, Norway

## Abstract

**Background:**

The clinical learning environment (CLE) is critical for developing the practical skills needed in healthcare professions. This study aimed to evaluate healthcare students' perceptions of the quality of the CLE using the Arabic version of the Clinical Learning Environment, Supervision and Nurse Teacher (ar. CLES + T) scale. The study also aimed to evaluate the tool's measurement invariance and compare perceptions among student groups.

**Methods:**

This cross-sectional study was carried out at two health education institutes in Morocco throughout the academic year 2018-2019 among 1550 undergraduate students who had just finished a clinical practicum in a hospital or primary healthcare facility. Data were gathered using the ar. CLES + T scale. Confirmatory factor analysis (CFA) and multigroup CFA were performed. The measurement invariance of the ar. CLES + T scale was assessed across gender, study year, and clinical practicum duration, using configural invariance, metric invariance, scalar invariance, and strict invariance. The *t*-test and analysis of variance were used to compare the mean scores of the student groups.

**Results:**

Students expressed positive perceptions toward the CLE. The “Pedagogical atmosphere on the ward” dimension scored the highest, while the “Role of the nurse teacher” dimension received the lowest scores. The measurement invariance of the ar. CLES + T scale by gender, study year, and clinical practicum duration was established. First-year students and those with an extended practicum period were the most satisfied.

**Conclusion:**

To promote effective learning in the clinical environment, nurse teachers might use innovative teaching approaches tailored to their evolving role in these settings. Moreover, extending the duration of clinical practicum can further enhance student learning outcomes.

## 1. Background

Health education is crucial for developing skilled individuals who can work as competent professionals. Clinical practicum is an essential component of health education programs, representing 40%–50% of European programs [1]. Clinical learning environment (CLE) has been widely recognized as a crucial learning area in health education because student learning occurs with patient contacts in a real clinical setting that no other solution, such as simulation, can fully replicate [2]. The factors of CLE have been identified to include the physical layout, psychosocial and interactional elements, organizational culture, and elements related to teaching and learning, such as effective teaching and student involvement [3].

Positive CLE can contribute to student professional development [1, 3], achievement of learning outcomes, increased self-confidence, and satisfaction with the healthcare profession [3]. However, negative CLE can lead to dissatisfaction with the field, increasing the risk of students' failure or even complete abandonment of the healthcare pursuit [1, 3] ultimately contributing to the global shortage of healthcare professionals [3]. Therefore, evaluating the CLE is an important step to identify areas for improvement and enhance student clinical learning experiences.

The quality of the supervisor–student relationship is the most important factor in shaping a student's professional development during clinical practicums [1]. There are various models of student supervision in the clinical context. Traditional methods of supervising students often relied on group-based approaches, whereas modern approaches prioritize personalized, one-on-one supervision [4]. A systematic review discovered that students were more satisfied with their supervisory relationships, which was linked to the students' experience with an individualized supervision model [5]. Students value confidential supervision meetings because it allows them to freely discuss the emotional challenges and reactions they encounter while caring for patients [6].

Moreover, students from six European countries valued the individualized supervision provided by their nurse teacher from the educational institute, as well as their support in enhancing learning and decreasing stress associated with clinical practicum [7]. Nevertheless, various studies have noted a general decline in the involvement of nurse teachers in clinical practice [6, 8, 9]. With the nurse education transition to higher education in many European countries, the clinical role of the nurse teacher has evolved from a competent practitioner to an intermediary between clinical settings and educational institutions [6, 9]. Furthermore, there is often confusion about what nurse teacher is supposed to do in clinical settings. Their role lacks a clear definition [1]. Previous research revealed negative perceptions among students regarding the clinical role of the nurse teacher [4, 5, 10–17] probably due to these changes. To address this issue and improve nurse teacher's cooperation with students, studies highlight the need to develop novel alternative approaches, such as digital educational technologies [7, 9, 18], to complement rather than substitute the physical presence of nurse teachers during placements [18]. Furthermore, the nurse teacher's clinical role should be revised to include clear responsibilities for student supervision during clinical practicum [7], distinct from the responsibilities of clinical supervisors [9].

In Morocco, research in nursing education is still in its earlier stages. So far, there have been few research types on the actual CLE. The primary focus has been on validating tools to assess the effectiveness of the CLE [19, 20]. Thus, we have not got a clear picture of Morocco's CLE until now. With the aim of providing an initial understanding of student-perceived CLE and suggesting improvements in clinical education, this study embarks on this exploration.

In 2013, the nursing education system in Morocco underwent substantial structural adjustments to align with the Bologna process, similar to other European Union countries [21]. This transition marked a shift from the traditional model, where state diplomas were awarded upon completion of vocational studies, to a university-based system emphasizing the attainment of Bachelor's, Master's, and Doctoral degrees [22]. Education occurs at the level of public institutions known as Higher Institutes of Nursing Professions and Health Techniques (ISPITS). These institutes, overseen by the Ministry of Health, provide higher education programs. The ISPITS includes 10 core institutes and affiliated units, strategically situated around the country.

The undergraduate degree program is available to students in five specific areas: nursing, midwifery, health techniques, rehabilitation, and medical–social assistance. Each pathway may comprise one or more options. Throughout this article, “healthcare students” refers to those pursuing any of these specialized fields. The license cycle lasts three years and consists of 2310 hours of theoretical and practical courses. Each academic year is divided into two semesters, each lasting 16 weeks. The license cycle in six semesters includes 38 training modules classified into main and complementary modules. Due to its importance, the clinical practicum is considered a main module.

Clinical practicums may begin as early as the first year for specific programs. In the second and third years, they become a substantial part of the curriculum, occupying over half (more than 51%) of the educational programs. At present, the duration of each practicum module is 120 hours. Previously, it spanned 160 hours longer but was adjusted to conform with the new educational standards implemented in higher education institutions. Clinical practicums can be full-time or part-time, and take place in public health facilities or other approved structures that provide students with learning opportunities.

To improve nursing education and ensure the transition to higher education, ISPITS has increased its recruitment of teachers with a doctorate in health sciences. Training for the PhD cycle focusing on nursing sciences is currently being prepared and will launch soon at the ISPITS. The recent creation of research structures would enable nursing research to take its rightful place within ISPITS though there are still challenges to be addressed [23]. Following a similar approach, the master's level training in nursing education has been extended to encompass all ISPITS, leading to a rise in the number of teachers qualified to serve as full-time faculty members at the education institutes. They deliver both theoretical and practical instructions in academic settings and supervise students in clinical placements relevant to their area of expertise.

In addition to their patient care responsibilities, the head nurses of the unit and the nursing staff also provide clinical supervision to students. Most European countries also use a similar supervision approach called the preceptor model [24]. Group supervision remains the traditional approach widely used in Moroccan settings. Nonetheless, there has been a significant shift toward personalized supervision in many European countries [4]. This individualized approach is recognized as the most impactful and critical model of supervision for students' professional development [1], as well as contributing to their satisfaction throughout clinical training [5, 13, 25–27].

This study aimed to evaluate Moroccan healthcare students' perceptions of the quality of the CLE using the validated ar. CLES + T scale. The study also aimed to assess the tool's measurement invariance and compare perceptions among student groups. Therefore, the study aimed to test the following hypothesis:

1. Healthcare students had positive perceptions of the CLE
2. The measurement invariance was established to compare students' perceptions by gender, student year, and clinical practicum duration
3. There were differences among students' perceptions based on gender, student year, and duration of clinical practicum

The findings from this study can inform strategies for enhancing clinical education and preparing competent professionals who can deliver high-quality patient care.

## 2. Methods

### 2.1. Study Design, Sample, and Settings

This cross-sectional study was conducted at two government health education institutes in Morocco during the academic year 2018–2019. Participants were included based on the following criteria: (1) undergraduate students in their first, second, or third year of a nursing or other health professions; (2) those who have recently completed a clinical practice course in a hospital ward or primary healthcare setting; and (3) those who provided informed consent. Exclusion criteria included students with no prior clinical experience and those who declined to participate. Using Cochran's formula [28, 29], this study requires at least 349 participants to achieve results with a 95% confidence level, a 5% margin of error, and an expected mean score and standard deviation of 3.26 (0.84), based on previous research [11] conducted in an Arab country similar to Morocco. However, to raise the power and precision of the study, all eligible students from the two institutions were included.

### 2.2. Instrument

This study used the CLES + T scale [1] to evaluate healthcare students' perceptions of the CLE. The CLES + T scale has been evaluated and validated in various studies. The scale demonstrated good reliability and validity in diverse contexts, including an Arabic version used in this study. The present study's Arabic CLES + T scale had good internal consistency, with a Cronbach's alpha coefficient of 0.93 for all subscales. A previous study has demonstrated the ar. CLES + T scale's validity for Moroccan healthcare students [19]. The ar. CLES + T scale comprises 34 items across five dimensions: pedagogical atmosphere on the ward, leadership style of the ward manager, premises of care on the ward, supervisory relationship, and role of the nurse teacher. Following their last clinical practicum, students completed a paper questionnaire at their institute, rating each item on a 5-point Likert scale. They also answered demographic and learning data questions (age, gender, degree program, student year, clinical placement, and duration of clinical practice).

### 2.3. Statistical Analysis

Descriptive statistics including frequencies and percentages were used to summarize demographic and learning data. The total mean score of the questionnaire was calculated as the mean of all item scores. Similarly, mean scores were calculated for each of the five dimensions by averaging the ratings of the corresponding items. Higher scores indicate more positive perceptions of the clinical learning environment, supervision, and the role of the nurse teacher.

To assess the measurement invariance of the Arabic CLES + T scale, confirmatory factor analysis was first performed to evaluate the model fit. If the factorial structure of a construct remains consistent across different subgroups, then measurement invariance can be assumed, indicating that the factor structure remains unchanged across these subgroups.

Gender, student year, and duration of clinical placement were the factors used to evaluate measurement invariance. The lavaan package was utilized to conduct a measurement invariance test through multiple-group factor analysis [30] for *R* statistics and weighted least squares mean and variance adjusted (WLSMV) estimation. The models proposed by Millsap and Yun-Tein [31] for ordered categorical variables were tested using the following procedure: configural invariance, which had no restrictions other than those needed for model identification, was tested first, followed by metric invariance where all factor loadings had to be similar. Scalar invariance was also tested, which required the threshold restriction already needed for model identification and was similar to weak invariance. Finally, strict invariance was tested, which involved restricting the unique variance to 1.

When conducting a measurement invariance test, the difference in the *χ*^2^ statistic is frequently employed, but due to its susceptibility to sample size, the primary indicator is the difference value of the comparative fit index (ΔCFI), which is a criterion of model fit [32]. To address the risk of overrejection with a small sample size, the difference values of the root mean square error of approximation (ΔRMSEA) and the standardized root mean square residual (ΔSRMR) are used as subcriteria. Chen [32] recommended the following cutoff criteria for noninvariance: ΔCFI ≤ −0.01, ΔRMSEA ≥ 0.01, and ΔSRMR ≥ 0.015. The authors noted that among the various indices used, CFI was the most highly consistent, whereas RMSEA tended to be more affected by factors, such as study population and model intricacy.

Students' perceptions were compared by gender, year of study, and duration of clinical practicum using either the *t*-test or analysis of variance (ANOVA), depending on the data distribution. Statistical analyses were conducted with IBM SPSS Statistics 23.0 and Amos 23.0 software.

### 2.4. Ethical Approval

The research protocol received ethical approval from the Mohammed V University of Rabat Ethics Committee (IRB: 69-2019). All participants received written information about the study's goals, confidentiality, anonymity, and voluntary contribution. Participants who signed the consent form, completed the questionnaire, and returned it were considered to have provided informed consent.

## 3. Results

### 3.1. Student Characteristics

The questionnaire was completed by 1550 students, achieving a 95% response rate. The majority of students (81%) were females, with a mean age ranging from 17 to 20 years (71% of respondents). Nursing students constituted a significant proportion of the participants (61%). The majority of respondents (82%) completed their clinical practicum at hospitals. Second-year students represented 45% of the respondents, followed by first-year students (32%) and third-year students (23%). The clinical practicum period lasted four weeks for almost half (46%) of the participants and the ward manager was most often (38%) responsible for student supervision, followed by the nurse and the specialized nurse. Group supervision was the most common type of supervision adopted (62%). More than half of the participants said they had never had an unscheduled meeting with their supervisor. Student characteristics are shown in Table 1.

### 3.2. Students' Perceptions of the Quality of the CLE

Students expressed overall positive perceptions toward their CLE, with an average score of 3.17 ± 0.76 on the total ar. CLES + T scale. Among the dimensions, “Pedagogical atmosphere on the ward” received the highest score (3.31 ± 0.82), indicating the most favorable perception. Conversely, the “Role of the nurse teacher” dimension received the lowest score (3.08 ± 1.03). Within this dimension, “Theory and practice integration of nurse teacher” was the most appreciated subdimension (3.31 ± 1.14), whereas “Relationship with mentor student and nurse teacher” was rated the least favorably (2.88 ± 1.19) (details are shown in Table 2).

### 3.3. Measurement Invariance Analysis of the ar. CLES + T Scale by Gender

Measurement invariance of the ar. CLES + T scale was established across gender-separated groups. Configural, metric (ΔCFI = 0.000, ΔRMSEA = 0.001, ΔSRMR = 0.001), scalar (ΔCFI = 0.000, ΔRMSEA = −0.003, ΔSRMR = 0.000), and strict invariance (ΔCFI = 0.000, ΔRMSEA = 0.000, ΔSRMR = 0.000) were all confirmed as reliable (see Table 3 for details).

### 3.4. Measurement Invariance Analysis of the ar. CLES + T Scale by Student Year

The ar. CLES + T scale demonstrated measurement invariance across three groups classified by year of study. Configural, metric (ΔCFI = −0.002, ΔRMSEA = 0.005, ΔSRMR = 0.004), scalar (ΔCFI = 0.000, ΔRMSEA = −0.003, ΔSRMR = −0.003), and strict invariance (ΔCFI = 0.000, ΔRMSEA = 0.000, ΔSRMR = 0.000) were all confirmed as reliable (see Table 3).

### 3.5. Measurement Invariance Analysis of the ar. CLES + T Scale by Clinical Practicum Duration

The ar. CLES + T scale demonstrated measurement invariance across three groups classified by clinical practicum duration (details in Table 3). All levels of invariance were confirmed as reliable, including configural, metric (ΔCFI = −0.002, ΔRMSEA = 0.003, ΔSRMR = 0.003), scalar (ΔCFI = 0.000, ΔRMSEA = −0.003, ΔSRMR = −0.002), and strict invariance (ΔCFI = 0.000, ΔRMSEA = 0.001, ΔSRMR = 0.000).

### 3.6. Students' Perceptions of Quality of the CLE by Gender

A gender difference was found in the “Pedagogical atmosphere on the ward” dimension. Male students perceived this dimension more favorably than female students (3.46 ± 0.79 vs 3.27 ± 0.83, *P* < 0.001). No significant gender differences were observed in other dimensions (see Table 4 for details).

### 3.7. Students' Perceptions of the Quality of the CLE by Student Year

First-year students reported the highest satisfaction with the total ar. CLES + T scale and its dimensions (3.33 ± 0.71), while third-year students reported the lowest (2.98 ± 0.75, *P* < 0.001). This indicates a significant difference in student satisfaction across year groups (details in Table 4).

### 3.8. Students' Perceptions of the Quality of the CLE by Clinical Practicum Duration

Students who completed a longer clinical practicum period reported significantly high mean scores compared to those who completed a shorter period regarding the “Pedagogical atmosphere on the ward” dimension (3.46 ± 0.83 vs 3.17 ± 0.74, *P* < 0.001), the “Leadership style of the ward manager” dimension (3.40 ± 0.98 vs 3.26 ± 0.92, *P* < 0.001), and the “Premises of care on the ward” dimension (3.35 ± 0.95 vs 3.09 ± 0.81, *P* < 0.001) (details in Table 4).

## 4. Discussion

Moroccan healthcare students generally held positive perceptions of their CLE, as evidenced by this study. However, their average ratings on the CLES + T scale fell below 4 out of 5, suggesting room for improvement in student satisfaction. This observation aligns with findings from other Arab countries like Saudi Arabia and Oman [10–12]. Notably, studies from European countries reported strong satisfaction (above 4) across all CLES + T components [5]. These comparisons highlight the need for enhancements to the Moroccan CLE to bridge the gap with other regions.

The study identified the “pedagogical atmosphere on the ward” as the most valued aspect of the CLE. This finding aligns with a wider review using the CLES + T scale, where all studies reported positive scores above 3 for this element [5]. This emphasizes the importance of fostering a supportive learning environment. Students thrive when surrounded by motivated and committed staff who actively engage and inspire them, allowing them to focus on their educational growth. Conversely, a negative environment can force students to prioritize their well-being, hindering their learning potential [1].

The study identified the “Role of the nurse teacher” as the least valued aspect of the CLE, consistent with findings from 16 other countries, although with inconsistencies [5]. This result might be related to the subdimension “relationship among student, mentor, and nurse teacher,” which received the lowest score among Moroccan students. Interestingly, a Slovakian study reported similar results [13], while Finnish students scored this subdimension the highest [6, 16]. Studies indicated that students' satisfaction increases with more frequent meetings with their supervisors and teachers [6, 10, 16, 26]. However, more than half of the participants in this study reported never having an unexpected meeting with their supervisors.

The nurse teacher's infrequent or missing interactions with the students could be related to the nurse teacher's reduced direct involvement in clinical areas, a trend seen in European countries following the transition to higher education [8]. This shift has moved the clinical role of the nurse teacher from a primarily hands-on practitioner to a mediator between educational institutions and healthcare providers [9]. Consequently, clinical teaching has diminished as teachers prioritize research and publication for career advancement within academic settings [1, 9]. Despite this, a well-structured CLE, combined with regular guidance from the clinical teacher, promoted active learning in students [12]. Obviously, physical presence in clinical settings may not always be necessary for a nurse teacher, but finding innovative ways to foster cooperation remains crucial [1]. A study conducted in nine European countries suggests that using e-communication strategies can significantly strengthen communication between clinical teachers and their students [9].

Another factor potentially contributing to the low score for the student–mentor–nurse teacher relationship is the inherent stress particularly when students are under observation or evaluation [13]. This could explain why Moroccan students gave the lowest score to the statement “In our common meeting, I felt that we are colleagues.”

Students perceived the “cooperation with ward staff” aspect of the nurse teacher role negatively. They were least likely to believe that the nurse teacher worked as a team member, aligning with findings from Sweden [33–35] and Finland [6, 16]. However, in Norway, a “dual preceptor team” model, where teachers and clinical staff collaborate, shows a positive impact on student learning [36]. These findings demonstrated that operating as a member of a nursing team does not effectively reflect the role of the nurse teachers in modern academic contexts of nursing education, especially given their limited participation in clinical practice [6, 33, 35]. Although Moroccan nurse teachers may have less direct clinical involvement, this does not diminish their clinical credibility, as students affirmed their ability to bridge the gap between theory and practice. This emphasizes that clinical credibility does not necessarily require constant clinical activity [34].

Similar to a previous Korean study [37], this study demonstrated the measurement invariance of the ar. CLES + T scale, ensuring it measures the same concepts across different student groups. The findings confirmed the tool's reliability for evaluating the quality of the CLE regardless of gender, student year, and clinical practicum duration. Measurement invariance across these groups was validated at the configural, metric, scalar, and strict invariance levels. Consequently, scores generated from this tool can be meaningfully compared across these three variables.

The present study found no significant correlation between gender and the overall mean score in line with previous European studies [4, 25, 33, 36]. However, a clear difference emerged in the way male and female students perceived the pedagogical atmosphere on the ward. Male students rated this dimension higher than female students in agreement with prior studies that identified notable differences between genders in the evaluation of certain CLES + T dimensions [11, 38–40]. These findings suggest the need for further investigation to understand the underlying reasons for this disparity and to develop strategies to improve the clinical experiences of female students.

This study showed a decline in student perceptions of the CLE as they progressed through their academic year. Conversely, an Ethiopian study revealed increased satisfaction with each year [40]. Other studies using the CLES + T tool found no significant differences in student experiences based on their year of study [4, 25]. Similar findings to the present study were reported in Cyprus and Koréa [26, 37]. Students' perspectives on the CLE might shift as they gain clinical experience. While initially drawn to the environment due to the novelty of learning new skills [41], their assessments might become more critical as they develop deeper understanding of fundamental concepts and their capacity for reflection improves [16]. Additionally, clinical supervisors and teachers might provide differing levels of supervision based on student experience, with a greater focus on first-year students who are novel to the environment [37]. This scenario could also apply in Morocco, where third-year students indicated dissatisfaction with the supervisory relationship and the role of the nurse teacher rather than the learning environment itself. Therefore, it is critical to inform supervisors and teachers about this phenomenon and encourage them to maintain strong supervision for students in their last year of training.

In the present study, satisfaction levels correlated with clinical practicum duration. Longer placements resulted in the most satisfied students. This outcome aligns with an earlier study across nine European countries [4]. Other studies reported the opposite, attributing this difference to the lower percentage of students who had completed extensive clinical training [10, 13, 42]. However, training to be a nurse requires sufficient time spent with patients [4]. Research suggests an ideal clinical placement period of approximately 7 weeks [43]. Therefore, the current length of clinical practicum for Moroccan students appears insufficient and should be extended to 7 weeks or more. This extended period would allow students to fully capitalize on the learning situations they find meaningful, sufficient, and diverse in the Moroccan healthcare settings.

### 4.1. Strengths and Limitations of the Study

The strength of this study is its pioneering evaluation of the Moroccan healthcare CLE from student perspectives, using an internationally validated instrument. However, a limitation is that our findings may not be generalizable to all of Morocco due to the use of convenience sampling, which does not fully represent the population of Moroccan public institutes. Additionally, the high gender ratio reflects the female-dominated nature of the healthcare profession in Morocco. Therefore, it is important to interpret our findings with caution, as men's and women's perceptions of the CLE may differ. Despite these limitations, our findings offer valuable preliminary insights into the CLE, potentially guiding decision-makers in making necessary improvements.

## 5. Conclusions and Implications

While Moroccan healthcare students generally held positive views of their CLE, there is room for improvement. Students identified the pedagogical atmosphere as crucial, yet expressed dissatisfaction with the reduced role of nurse teachers in clinical contexts. Scholarly literature offers innovative approaches to clinical education that could be implemented in Morocco. Such approaches aim to improve student learning while reducing the need for frequent face-to-face interactions with nurse teachers in clinical settings. Studies suggest the potential of using e-communication tools, such as e-mail, mobile solutions, and virtual learning environments, to strengthen teacher-student relationships during placements [6, 9, 44]. Nonetheless, evidence suggests that e-communication cannot fully replace face-to-face contact [9, 44]. Another pedagogical alternative is for the nurse teacher to focus on simulated learning in academic environment [6]. This approach aligns well with Morocco's recent advancements in simulation training, marked by the creation of simulation centers and nurse teacher training initiatives in nursing education institutes.

The ar. CLES + T scale could be used to evaluate the quality of the CLE among Moroccan healthcare students across variables like gender, study year, and clinical practicum duration. Evaluating the invariance of this tool across different variables is crucial to identify factors influencing clinical learning and guide targeted improvements.

First-year students were the most satisfied with the CLE. This suggests that nursing supervisors and teachers may need to adjust their clinical teaching methods to better address the evolving learning needs of students, particularly in their final year. Longer practicum periods led to increased student satisfaction. Therefore, extending the practicum duration to at least 7 weeks could be a valuable strategy to enhance student learning outcomes.

The current study provides an initial exploration of the CLE from the perspective of healthcare students in two Moroccan institutes. For a comprehensive understanding of the CLE across the country, conducting further studies with diverse student populations from diverse clinical settings is recommended. The ultimate goal is to improve the CLE and prepare competent professionals capable of delivering high-quality patient care.

## Figures and Tables

**Table 1:** Participants' characteristics and supervision parameters (*N* = 1550).

Characteristics	No. (%)
Age (year)	
17–20	1097 (70.7)
21–24	434 (28)
25–46	19 (1.3)
Gender	
Female	1250 (80.6)
Male	300 (19.4)
Degree program	
Nursing	953 (61.5)
Other health professions	597 (38.5)
Year of study	
First	499 (32.2)
Second	697 (45.0)
Third	354 (22.8)
Clinical placement	
Hospital	1266 (81.7)
Primary healthcare	284 (18.3)
Clinical practicum duration	
2 weeks or less	299 (19.3)
3 weeks	532 (34.3)
4 weeks	719 (46.4)
Occupational title of the supervisor	
Nurse	283 (18.3)
Nurse specialist	266 (17.2)
Ward manager	587 (37.9)
Other	414 (26.7)
Occurrence of supervision	
No supervisor nominated	25 (1.6)
Bad relationship with a named supervisor	5 (0.3)
Supervisor changed during the placement	10 (0.6)
Supervisor changed between shifts or placements	532 (34.3)
Supervisor had several students	958 (61.8)
Good relations with a named supervisor	20 (1.3)
Frequency of separate, unscheduled private meetings with the supervisor	
Not at all	831 (53.6)
Once or twice during the course	272 (17.5)
Less than once a week	126 (8.1)
About once a week	108 (7.0)
More often	213 (13.7)

Data are presented as number (%).

**Table 2:** Mean scores of total ar. CLES + T scale and dimensions (*N* = 1550).

	Mean ± SD
Total ar. CLES + T	3.17 ± 0.76
Dimensions	
(1) Pedagogical atmosphere on the ward	3.31 ± 0.82
(2) Leadership style of the ward manager	3.29 ± 0.99
(3) Premises of care on the ward	3.24 ± 0.93
(4) Supervisory relationship	3.18 ± 1.01
(5) Role of the nurse teacher	3.08 ± 1.03
(i) Theory and practice integration of nurse teacher	3.31 ± 1.14
(ii) Cooperation with ward staff of nurse teacher	3.04 ± 1.17
(iii) Relationship with mentor student and nurse teacher	2.88 ± 1.19

Data are presented as mean (standard deviation). ar. CLES + T: Arabic version of clinical learning environment, supervision, and nurse teacher scale.

**Table 3:** Analysis of measurement invariance of the ar. CLES + T scale according to gender, student year, and clinical practicum duration (*N* = 1550).

Group	Invariance model	*χ* ^2^	df	CFI	SRMR	RMSEA	95% CI	ΔCFI	ΔSRMR	ΔRMSEA
Gender	Configural	2561.05	1028	0.992	0.044	0.044	0.042–0.046			
	Metric	2742.11	1057	0.992	0.045	0.045	0.043–0.047	0.000	0.001	0.001
	Scalar	2723.80	1154	0.992	0.045	0.042	0.040–0.044	0.000	0.000	−0.003
	Strict	2732.55	1157	0.992	0.045	0.042	0.040–0.044	0.000	0.000	0.000

Student year	Configural	2950.19	1542	0.993	0.048	0.042	0.040–0.044			
	Metric	3386.10	1600	0.991	0.052	0.047	0.044–0.049	−0.002	0.004	0.005
	Scalar	3600.94	1794	0.991	0.049	0.044	0.042–0.046	0.000	−0.003	−0.003
	Strict	3634.30	1800	0.991	0.049	0.044	0.042–0.047	0.000	0.000	0.000

Clinical practicum duration	Configural	3027.28	1542	0.993	0.049	0.043	0.041–0.045			
	Metric	3382.18	1600	0.991	0.052	0.046	0.044–0.049	−0.002	0.003	0.003
	Scalar	3517.88	1794	0.991	0.050	0.043	0.041–0.045	0.000	−0.002	−0.003
	Strict	3571.01	1800	0.991	0.050	0.044	0.042–0.046	0.000	0.000	0.001

*χ* ^2^: chi-square value, df: degree of freedom, CFI: comparative fit index, SRMR: standardized root mean residual, RMSEA: root mean square error of approximation, CI: confidence interval, Δ: difference of value.

**Table 4:** Comparison between mean scores of total ar. CLES + T scale and dimensions among gender, student year, and clinical practicum duration groups of students (*N* = 1550).

Factor	Total ar. CLES + T	Pedagogical atmosphere on the ward	Leadership style of the ward manager	Premises of care on the ward	Supervisory relationship	Role of the nurse teacher
Gender						
Male	3.21 (0.76)	3.46 (0.79)	3.25 (1.00)	3.3 (0.87)	3.27 (0.98)	3.10 (1.03)
Female	3.16 (0.76)	3.27 (0.83)	3.31 (0.99)	3.22 (0.94)	3.16 (1.02)	3.07 (1.03)
*T*-test	−1.13	−3.66	0.92	−1.52	−1.64	−0.44
*P* value	0.259	<0.001	0.357	0.129	0.100	0.654
Student year						
First	3.33 (0.71)	3.38 (0.80)	3.41 (1.00)	3.32 (0.92)	3.30 (1.00)	3.31 (0.91)
Second	3.14 (0.77)	3.34 (0.80)	3.28 (1.01)	3.27 (0.91)	3.20 (1.00)	3.01 (1.08)
Third	2.98 (0.75)	3.14 (0.86)	3.16 (0.94)	3.05 (0.75)	2.97 (1.01)	2.89 (1.02)
*F* statistics	22.69	9.73	6.35	9.71	11.32	20.87
*P* value	<0.001	<0.001	0.002	<0.001	<0.001	<0.001
Practicum duration						
80 h	3.06 (0.71)	3.17 (0.74)	3.26 (0.92)	3.09 (0.81)	3.18 (0.93)	2.95 (1.05)
120 h	3.12 (0.73)	3.18 (0.83)	3.17 (1.03)	3.16 (0.93)	3.11 (1.05)	3.09 (0.96)
160 h	3.24 (0.79)	3.46 (0.83)	3.40 (0.98)	3.35 (0.95)	3.23 (1.02)	3.12 (1.06)
*F* statistics	7.07	23.30	8.74	11.38	2.06	3.02
*P* value	0.001	<0.001	<0.001	<0.001	0.127	0.049

Data are presented as mean (standard deviation).

## Data Availability

The corresponding author will share the datasets used in this study upon reasonable request.
